# Analysis of clinical and genetic features in a pediatric patient with Kartagener syndrome caused by compound heterozygous mutations in the *DNAH5* gene: a case study and literature review

**DOI:** 10.3389/fmed.2025.1513370

**Published:** 2025-03-28

**Authors:** Jingli Zhang, Longfei Gao, Yu Xing, HuiFang Wu, Xiaojuan Liu, Yingqian Zhang

**Affiliations:** Third Department of Respiratory, Hebei Children's Hospital, Shijiazhuang, China

**Keywords:** primary ciliary dyskinesia, Kartagener syndrome, ciliary function, *situs inversus*, genetic mutations, *DNAH5*

## Abstract

Kartagener syndrome (KS), a subtype of primary ciliary dyskinesia (PCD), is a rare genetic disorder characterized by *situs inversus*, chronic sinusitis, bronchiectasis, recurrent respiratory infections, and impaired ciliary function. It is diagnosed through physical examination, imaging techniques such as computed tomography (CT), nasal nitric oxide measurement, genetic testing, and pulmonary function tests. We present a case study of a 15-year-and-11-month-old male patient with KS complicated by sinusitis, secretory otitis media, and bronchiectasis. The patient exhibited *situs inversus totalis*, affecting the lungs, heart, and abdominal organs. Treatment included antibiotics for infection, mucolytics, and pulmonary rehabilitation. Postural drainage and bronchoscopy were performed for lung lavage. Following treatment, the patient’s respiratory symptoms improved, and lung function tests showed improvement. A literature review identified a high prevalence of lung and heart transpositions in Chinese patients with PCD, while abdominal organ transposition was less commonly reported. Genetic analysis revealed compound heterozygous mutations in the *DNAH5* gene, specifically c.12279 + 1 G > A (exon 71, NM_001369) and c.9457 C > T (exon 56, NM_001369), including the newly discovered variant c.9457 C > T (exon 56, NM_001369). This novel mutation expands the genetic landscape associated with KS, providing further insights into the underlying genetic basis of the condition. The study emphasizes the clinical features, the limited reporting of abdominal organ transposition, the genetic basis, and the treatment of KS, thereby contributing to the understanding and management of this condition.

## 1 Introduction

Kartagener syndrome (KS), a subtype of primary ciliary dyskinesia (PCD), is a rare genetic disorder characterized by *situs inversus*, chronic sinusitis, bronchiectasis, recurrent respiratory infections, and impaired motile ciliary function. It is diagnosed through physical examination, imaging techniques such as computed tomography (CT), nasal nitric oxide measurement, genetic testing, and pulmonary function tests (1).

Motile cilia are microscopic, hair-like structures found on the surface of various cells in the body, including those in the respiratory tract, reproductive system, and other organs. They play a crucial role in mucus movement, pathogen clearance, and the coordination of various cellular processes. Defects in motile ciliary structure or function can lead to a range of clinical manifestations, including chronic respiratory infections, bronchiectasis, sinusitis, and *situs inversus totalis* (2, 3).

*Situs inversus totalis* is a condition in which the organs of the chest and abdomen are mirrored or transposed from their usual positions. This occurs due to a developmental abnormality during embryogenesis and is seen in approximately 50% of individuals with PCD (4, 5). This condition arises from the dysfunction of nodal cilia, which disrupts left–right asymmetry during embryonic development, resulting in conditions such as *situs inversus*. Nodal cilia (6) generate a directed flow of fluid within the node region, initiating a signaling cascade that ultimately orchestrates the accurate alignment of internal organs along the left–right axis. Any malfunction or irregularity in nodal cilia can disrupt this intricate process, leading to *situs inversus totalis,* where the typical left–right organ positioning is reversed.

The clinical presentation of KS is diverse, but the most common features include chronic respiratory symptoms such as chronic cough, recurrent respiratory infections, chronic secretory otitis media, and infertility. Sinusitis is also a common manifestation, leading to chronic nasal congestion, sinus pressure, and nasal discharge. Additionally, *situs inversus totalis* affects the positioning of the heart, lungs, and abdominal organs, although the extent of organ transposition can vary among individuals.

Diagnosing KS requires a comprehensive evaluation, including clinical assessment, imaging analysis, and functional tests. Chest X-rays, CT scans, and echocardiography are commonly performed to assess the presence of *situs inversus totalis* and associated respiratory and cardiac abnormalities. High-resolution electron microscopy carried out on airway respiratory cilia can provide insights into the structural abnormalities of cilia, while genetic testing plays a crucial role in identifying specific gene mutations associated with the condition.

Management of KS focuses on symptomatic treatment, prevention of respiratory infections, and promotion of airway clearance. Antibiotics are prescribed to treat infections, while mucolytics and pulmonary physiotherapy techniques aid in mucus clearance. Physical therapy techniques, such as postural drainage and bronchoscopy with lavage, can also be used to facilitate the removal of secretions from the airways.

In this case study, we present a 15-year-and- 11-month-old male patient with KS, highlighting the clinical features, diagnostic process, genetic findings, and treatment outcomes. The identification of a novel mutation in the *DNAH5* gene expands our understanding of the genetic basis of KS. This report contributes to the existing literature on KS, emphasizing the importance of early recognition, accurate diagnosis, and appropriate management of this rare genetic disorder.

## 2 Presentation

The patient, a 15-year-and-11-month-old male, had a history of recurrent respiratory infections since the fourth day after birth, characterized by cough, sputum production, nasal congestion, and yellow nasal discharge without wheezing. The child received repeated antimicrobial treatments locally, resulting in symptom improvement. However, these symptoms have persisted and gradually worsened over the past 14 years, accompanied by decreased exercise tolerance and occasional self-resolving dizziness. At the age of 14 years and 8 months, the patient began experiencing chest tightness, which significantly worsened when lying flat at night but improved when in an upright position. The patient had previously been diagnosed with bronchiectasis and sinusitis. He is the second child of the family, born at full term with normal birth weight and without complications. Both his parents and his siblings are in good health, and there is no family history of genetic or infectious diseases.

On admission, the patient’s vital signs—temperature, pulse rate, respiratory rate, and blood pressure—were within normal ranges. Physical examination revealed that the patient appeared alert and responsive, with no cyanosis observed on the lips. An examination of the throat revealed congestion, and the tonsils were moderately enlarged (grade II). The chest exhibited deformities characterized by asymmetric thoracic structures and localized protrusion on the left side. No nasal flaring was observed, and the three-concave sign was negative. Auscultation of the lungs revealed wet rales bilaterally, and digital clubbing was present.

Laboratory tests showed a mild elevation in white blood cell count during routine blood tests, and C-reactive protein levels were also detected. Other laboratory parameters, such as liver and kidney function, electrolytes, serum proteins, coagulation function, ferritin, and total T lymphocyte analysis, were generally within normal ranges. Tests for hepatitis viruses A, B, C, syphilis spirochetes, and human immunodeficiency virus were all negative. Immunoglobulin A was elevated at 4.74 g/L (normal range: 0.47–2.49 g/L), and immunoglobulin M was elevated at 2.21 g/L (normal range: 0.15–1.88 g/L), while immunoglobulin G levels were normal. Sputum culture confirmed the presence of *Pseudomonas aeruginosa*. Lung function testing revealed a mixed ventilatory function disorder characterized by significant reductions in lung capacity and expiratory flow rates. The fractional exhaled nitric oxide (FeNO) level was normal.

**Figure 1:**
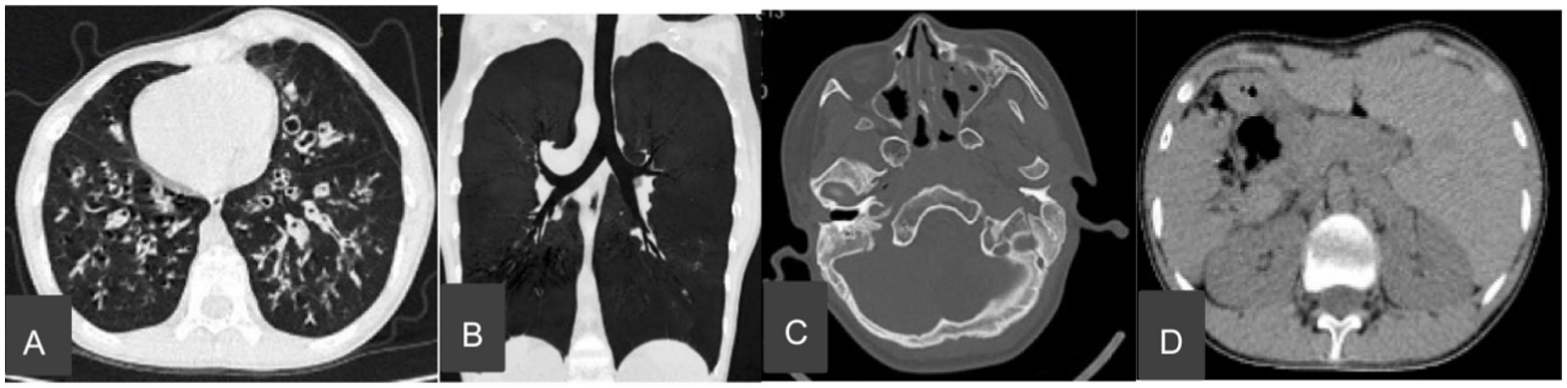
Computed tomography (CT) image of chest and nasal. **(A)** Bronchiectasis and bronchiolitis. **(B)** *Situs inversus totalis*. **(C)** Sinusitis. **(D)** Abdominal organ displacement.

**Figure 2:**
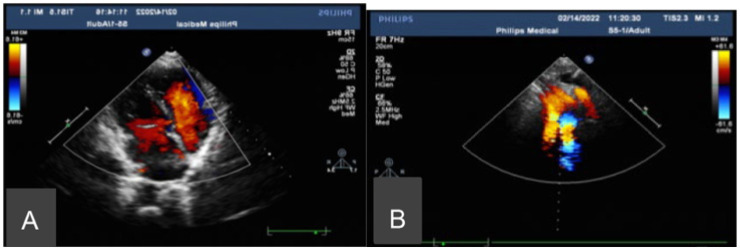
Echocardiography. **(A)** Dextrocardia with *situs inversus*. **(B)** A right-sided aortic arch.

Chest CT showed bronchiectasis with infection in both lungs (Figure 1A), accompanied by *situs inversus totalis* (Figure 1B) and sinusitis (Figure 1C). Abdominal CT confirmed *situs inversus* (Figure 1D). Echocardiography demonstrated dextrocardia with *situs inversus* (Figure 2A) and a right-sided aortic arch (Figure 2B). Otoscopy revealed congestion in the left tympanic membrane and cloudiness in the right with unclear landmarks.

Preliminary diagnosis: pneumonia, bronchiectasis, and associated visceral *situs inversus*. The treatment plan included ceftazidime for infection, acetylcysteine for mucolysis, and mechanical assistance for sputum clearance. An ENT specialist consultation revealed bilateral abundant purulent nasal discharge, hypertrophied inferior turbinates, and signs of rhinitis and sinusitis. Aggressive anti-inflammatory and decongestant treatments were initiated, such as saline nasal irrigation and nasal steroid sprays. Otoscopic examination indicated intact bilateral tympanic membranes, with congestion in the left and opacity in the right, suggestive of acute otitis media (left) and serous otitis media (right). Ofloxacin ear drops were administered to the left ear.

Following the treatment, the child’s cough improved, and there was a noticeable change in the color of sputum and nasal discharge, transitioning from yellow to white. Subsequent lung function tests revealed an improvement compared to previous results. However, the cough with sputum and nasal discharge persisted. Due to the patient’s history of recurrent respiratory infections, rhinitis, and sinusitis since childhood, along with worsening symptoms, decreased exercise tolerance, and occasional dizziness, the pulmonary CT scan findings of bronchiectasis and visceral *situs inversus* raised the suspicion of KS.

**Figure 3:**
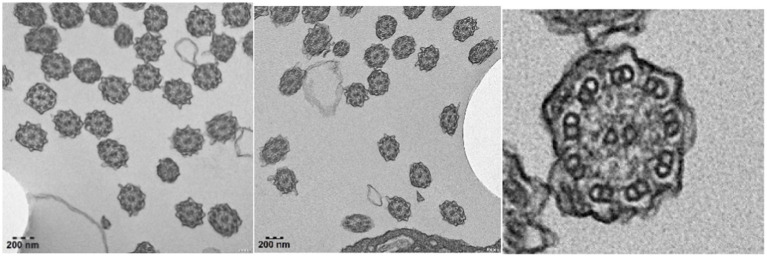
Electron microscopy imaging revealed respiratory cilia with a notable absence of outer dynein arms.

Bronchoscopy lavage treatment was performed, revealing a significant amount of yellow-white thread-like secretions in the airway, along with fishbone-like changes. Microscopic examination of the bronchial mucosa showed thinning and shedding of epithelium, fibrous hyperplasia underneath, mild infiltration of inflammatory cells, and fibrin exudation. Imunohistochemistry of the bronchial mucosa showed positive staining for cytokeratin, smooth muscle actin, and vimentin, while showing negative results for acid-fast and periodic acid-Schiff staining, indicating the presence of epithelial and smooth muscle cells, mesenchymal cells, and the absence of acid-fast bacteria or glycogen in the tissue. Electron microscopy examination of cilia structure revealed a clear 9 + 2 microtubule structure in some cilia cross-sections, with a significant absence of outer dynein arms in all evaluable cilia (Figure 3).

**Figure 4:**
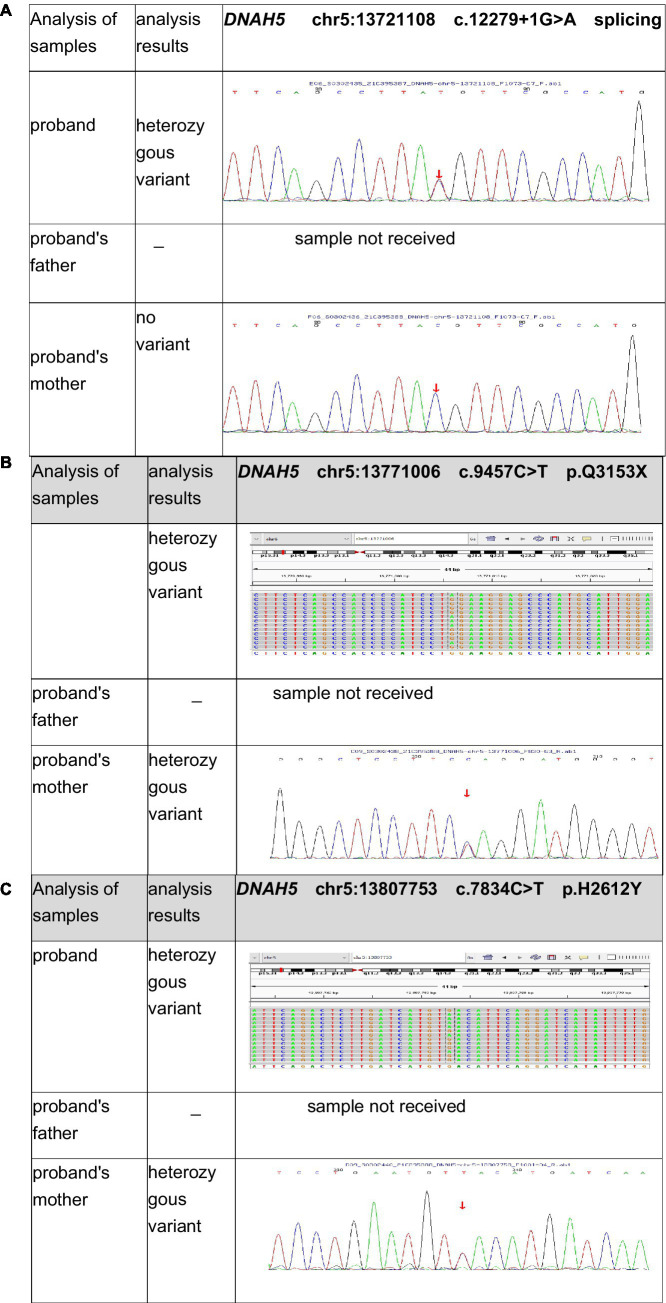
Test results for the patient’s first and second generation revealed the following mutations in *DNAH5* (NM_001369): **(A)** Exon 71: c.12279 + 1 G > A. **(B)** Exon 56: c.9457 C > T. **(C)** Exon 47: c.7834C > T.

Furthermore, genetic testing was performed. Ethical approval was obtained from our hospital, and informed consent was obtained from the parents. Peripheral blood samples were collected from both the patient and his mother. The child’s father was unable to provide a blood sample due to COVID-19 prevention and control measures. He is working in another city and is unable to return for the blood collection. The samples were sent to the Medical Laboratory of Mygenostics (Mygenostics Co. Ltd.) for whole-exome sequencing. Pathogenicity analysis was conducted according to the guidelines of the American College of Medical Genetics and Genomics (ACMG). The following variants were identified in the *DNAH5 (NM_001369) gene* of the child (Table 1; Figure 4).

**Table 1:** Gene variants for the affected child.

Gene	Chromosomal position	Transcript exon	Nucleotide amino acid	Homozygous/heterozygous	Normal population frequency	Prediction	ACMG pathogenicity analysis	Phenotype (inheritance)	Source of variation
*DNAH5*	chr5:13721108	NM_0013 69; exon71	c.12279 + 1G > A (splicing)	Het	0.0000119	-	Pathogenic	Primary ciliary dyskinesia type 3 (AR)	Father’s sample not received
*DNAH5*	chr5:13771006	NM_0013 69; exon56	c.9457C > T (p.Q3153X)	Het	-	-	Likely pathogenic	Primary ciliary dyskinesia type 3 (AR)	Mother
*DNAH5*	chr5:13807753	NM_0013 69; exon47	c.7834C > T (p.H2612Y)	Het	0.000008	B	Uncertain	Primary ciliary dyskinesia type 3 (AR)	Mother

Prediction: protein function prediction software REVEL, D, predicted as damaging, LD predicted as likely damaging, B predicted as benign, −: unknown.

Based on the diagnostic analysis provided, the child was ultimately diagnosed with Kartagener syndrome. Specifically, the child exhibited *situs inversus totalis*, affecting the lungs, heart, and abdominal organs.

Exon 71: c.12279 + 1 G > A (Figure 4A), resulting in an amino acid splice site mutation. This variant was preliminarily classified as a pathogenic variant with strong evidence (PVS1 + PM2_Supporting+PM3_Strong).
Exon 56: c.9457 C > T (Figure 4B), resulting in an amino acid change p.Q3153X, a nonsense mutation. This variant was preliminarily classified as a likely pathogenic variant with supporting evidence (PVS1 + PM2_Supporting). No relevant reports on this variant were found in the literature databases.
Exon 47: c.7834C > T (Figure 4C), resulting in an amino acid change p.H2612Y, a missense mutation. This variant was preliminarily classified as a variant of uncertain clinical significance with supporting evidence (PM2_Supporting+BP4).

Sanger sequencing was performed for validation. Parental origin analysis revealed that the child’s mother carried heterozygous variants at c.9457C > T (p.Q3153X) and c.7834C > T (p.H2612Y), while the variant at 12279 + 1G > A (splicing) was not detected. The child’s father was not sampled. Consequently, the child was found to have compound heterozygous mutations in the *DNAH5* (NM_001369) gene: exon 71:c.12279 + 1G > A (splicing) and exon 56:c.9457 C > T (p.Q3153X).

## 3 Literature review

In this paper, we explored the presentation and clinical characteristics of PCD or KS in China. We conducted a literature review using the following keywords in the Chinese databases Wanfang and CNKI: “Primary Ciliary Dyskinesia,” “Kartagener Syndrome,” “*DNAH5*,” and “China.” We selected cases with visceral transposition from the literature search. In the PubMed database, we used the keywords “primary ciliary dyskinesia,” “Kartagener syndrome,” “*situs inversus*,” “*DNAH5*,” and “China” for the search.

Based on our literature review, we identified 15 relevant articles involving 17 patients. The articles selected pertain to PCD or KS in China, specifically focusing on cases with visceral transposition and *DNAH5* mutations. Among these, 16 patients (94.1%) were reported to have *situs inversus* of the lungs, while information on lung *situs inversus* was not provided for 1 patient (5.9%). Additionally, all 17 patients (100%) had *situs inversus* of the heart. However, only 6 patients (35.3%) were reported to have abdominal organ transposition, while information on abdominal organ transposition was not mentioned for 11 patients (64.7%).

## 4 Discussion

KS, also known as a subtype of PCD, is a rare autosomal recessive disorder characterized by chronic sinusitis, bronchiectasis, and *situs inversus totalis* (1, 4). The patient in this case study exhibited typical clinical features of KS, including recurrent respiratory infections, chronic cough, nasal congestion, and bronchiectasis. The presence of *situs inversus totalis*, dextrocardia, and right-sided aortic arch further supported the diagnosis. Additionally, physical examination findings, such as digital clubbing, wet rales on lung auscultation, and chest deformities, reinforced the presence of chronic respiratory disease.

These research findings (7, 8) highlight a high prevalence of lung and heart transpositions in Chinese patients with PCD or KS. However, it should be noted that while abdominal organ transposition appears to be less commonly reported in these patients, it does not necessarily indicate a low incidence of the condition. The lack of mention in the article could suggest insufficient attention from healthcare professionals toward abdominal organ transposition in KS. Further studies and case reports are needed to fully understand the prevalence and clinical implications of abdominal organ transposition in these patients. By increasing awareness of this aspect, clinicians can improve the understanding and treatment provided to individuals with KS.

KS is primarily caused by defects in the structure or function of motile cilia (1), which can result from mutations in various genes (9, 10). Among these genes, *DNAH5* plays a crucial role in ciliary function (11). The *DNAH5* gene encodes dynein axonemal heavy chain 5, a protein crucial for the structure and function of motile cilia (12). Mutations in the *DNAH5* gene disrupt the normal ciliary function, leading to impaired mucociliary clearance and subsequent respiratory and reproductive tract abnormalities seen in KS (13).

In the present case, whole-exome sequencing identified compound heterozygous mutations in the *DNAH5* gene. The variants identified, including the newly discovered mutation in exon 56 (c.9457 C > T), contribute to the development of KS. These mutations were classified based on the ACMG guidelines, with the splice site mutation in exon 71 (c.12279 + 1 G > A) being classified as pathogenic and the mutation in exon 56 (c.9457 C > T) as likely pathogenic. Understanding the genetic basis of KS, particularly the role of *DNAH5* gene mutations, is crucial for accurate diagnosis, genetic counseling, and potential targeted therapies in the future.

In this study, electron microscopy analysis revealed significant structural abnormalities in the ciliated epithelial cells of patients with PCD. Specifically, there was a notable absence of outer dynein arms in all evaluated cilia, which is consistent with previous reports linking PCD to the loss of outer dynein arms. This observation further supports the understanding that the absence of outer dynein arms impairs the coordinated movement of cilia, leading to compromised mucociliary clearance and the accumulation of mucus in the respiratory tract. Consequently, our findings suggest a potential pathological basis for bronchiectasis and chronic respiratory symptoms in PCD. Based on the literature (6, 7), *DNAH5* mutations are associated with cilia structural abnormalities, such as outer dynein arm loss, consistent with our observations. However, further studies are needed to confirm these findings.

The treatment approach for KS focuses on managing respiratory symptoms, preventing infections, and improving mucociliary clearance (14). In the present case, the patient received cephalosporin antibiotics to address the *Pseudomonas aeruginosa* infection, acetylcysteine for mucolysis, and pulmonary rehabilitation. Postural drainage and bronchoscopy with lavage were performed to facilitate clearance of secretions from the airways. These interventions resulted in clinical improvement, with the resolution of cough and improvements in sputum and nasal discharge. In advanced stages of PCD, lung transplantation can be considered. Marro et al. (15) described the outcomes of the largest lung transplant population for PCD and Kartagener Syndrome, suggesting that lung transplantation is an acceptable treatment option in this population.

The patient’s mother expressed dissatisfaction with the previous treatments for recurrent respiratory infections. They sought care at a leading children’s hospital, where, despite efforts, the child’s condition persisted. While a definitive diagnosis was made, the parents felt that a cure had not been achieved. They believed the worsening of the child’s condition was linked to factors at the boarding school, where the child refrained from coughing forcefully, resulting in mucus retention and bronchiectasis. Following effective communication with the doctors, the parents and child embraced pulmonary rehabilitation and preventive measures, leading to a reduction in hospitalizations. Psychological counseling supported the child’s engagement in outdoor activities.

In conclusion, this case report highlights the clinical features, the underrepresentation of abdominal organ transposition, the genetic basis, and the treatment of KS in a 15-year-and-11-month-old male patient. The identification of compound heterozygous mutations in the *DNAH5* gene further elucidates the pathogenesis of the syndrome. By increasing awareness of KS and its various aspects, clinicians can improve diagnosis, management, genetic counseling, and potential therapeutic approaches for affected patients. Parental feedback underscores the importance of ongoing communication, psychological support, and societal assistance for patients with chronic illnesses.

## Data Availability

The original contributions presented in the study are included in the article/supplementary material, further inquiries can be directed to the corresponding author.
